# Author Correction: Butyrate extends health and lifespan in mice with mitochondrial deficiency

**DOI:** 10.1038/s41467-026-76269-x

**Published:** 2026-08-10

**Authors:** Enrique Gabandé-Rodríguez, Manuel M. Gómez de las Heras, Pablo Ramírez-Ruiz de Erenchun, Carolina Simó, Virginia García-Cañas, Naohiro Inohara, Inés Berenguer-López, Violeta Enríquez-Zarralanga, Álvaro Fernández-Almeida, Jorge Oller, Gonzalo Soto-Heredero, Elisa Carrasco, Cristina Vázquez-Muñoz, Sandra Delgado-Pulido, José Ignacio Escrig-Larena, Isaac Francos-Quijorna, Raquel Justo-Méndez, Juan Francisco Aranda, Joanna Poulton, Ana Victoria Lechuga-Vieco, José Antonio Enríquez, Gabriel Núñez, María Mittelbrunn

**Affiliations:** 1 Tissue and Organ Homeostasis Program, Centro de Biología Molecular Severo Ochoa (CBM), Consejo Superior de Investigaciones Científicas (CSIC) - Universidad Autónoma de Madrid (UAM), Madrid, Spain; 2 Departament of Molecular Biology, Faculty of Science, Universidad Autónoma de Madrid (UAM), Madrid, Spain; 3 Molecular Nutrition and Metabolism, Institute of Food Science Research (CIAL), Consejo Superior de Investigaciones Científicas (CSIC) - Universidad Autónoma de Madrid (UAM), Madrid, Spain; 4 Department of Pathology and Rogel Cancer Center, University of Michigan Medical School, Ann Arbor, MI USA; 5 Physiological and Pathological Processes Program, Centro de Biología Molecular Severo Ochoa (CBM), Consejo Superior de Investigaciones Científicas (CSIC) - Universidad Autónoma de Madrid (UAM), Madrid, Spain; 6 Laboratory of Vascular Pathology, IIS-Fundación Jiménez Díaz, Madrid, Spain; 7 Centro de Investigación Biomédica en Red de Enfermedades Cardiovasculares (CIBERCV), Faculty of Medicine and Biomedicine, Universidad Alfonso X El Sabio, Madrid, Spain; 8 Department of Biology, Faculty of Sciences, Universidad Autónoma de Madrid (UAM), Madrid, Spain; 9 Instituto Universitario de Biología Molecular-IUBM, Universidad Autónoma de Madrid (UAM), Madrid, Spain; 10 Instituto Ramón y Cajal de Investigación Sanitaria (IRYCIS), Madrid, Spain; 11 Cardiovascular Regeneration Program, Centro Nacional de Investigaciones Cardiovasculares (CNIC), Madrid, Spain; 12 Department of Genetics, Physiology and Microbiology, Faculty of Biological Sciences, Complutense University of Madrid (UCM), Madrid, Spain; 13 Nuffield Department of Women’s and Reproductive Health, University of Oxford, Oxford, UK; 14 Institute for Research in Biomedicine (IRB Barcelona), The Barcelona Institute of Science and Technology, Barcelona, Catalonia Spain; 15 Centro de Investigación Biomédica en Red de Fragilidad y Envejecimiento Saludable (CIBERFES), Madrid, Spain; 16 Center for Infectious Disease Education and Research (CiDER); Osaka University, Suita, Osaka Japan

**Keywords:** Mitochondria, Microbial communities

Correction to: *Nature Communications* 10.1038/s41467-026-70547-4, published online 13 March 2026

In this article there was an error in Fig. s3k and the associated source data. Specifically, the data corresponding to Tjp1 and Tff3 in ITfamKO mice were inadvertently duplicated. The SI file and Source Data file has been corrected.

