# Supplementary material for: Butyrate extends health and lifespan in mice with mitochondrial deficiency
Source: Nat Commun. 2026 Mar 13;17:3909. doi: 10.1038/s41467-026-70547-4 (PMC13129094; doi:10.1038/s41467-026-70547-4)

Supplementary Information for:

Butyrate extends health and lifespan in mice with mitochondrial deficiency

Enrique Gabandé-Rodríguez[†], Manuel M. Gómez de las Heras[†], Pablo Ramírez-Ruiz de Erenchun, Carolina Simó, Virginia García-Cañas, Naohiro Inohara, Inés Berenguer-López, Violeta Enríquez-Zarralanga, Álvaro Fernández-Almeida, Jorge Oller, Gonzalo Soto-Heredero, Elisa Carrasco, Cristina Vázquez-Muñoz, Sandra Delgado-Pulido, José Ignacio Escrig-Larena, Isaac Francos-Quijorna, Raquel Justo-Méndez, Juan Francisco Aranda, Joanna Poulton, Ana Victoria Lechuga-Vieco, José Antonio Enríquez, Gabriel Núñez and María Mittelbrunn[*]

[†] These authors contributed equally to this work

[*] Corresponding author. Email: mmittelbrunn@cbm.csic.es

This PDF file includes:

Figures S1 to S10.

Tables S1 and S2.

Uncropped scans of blots shown in Supplementary Figures.

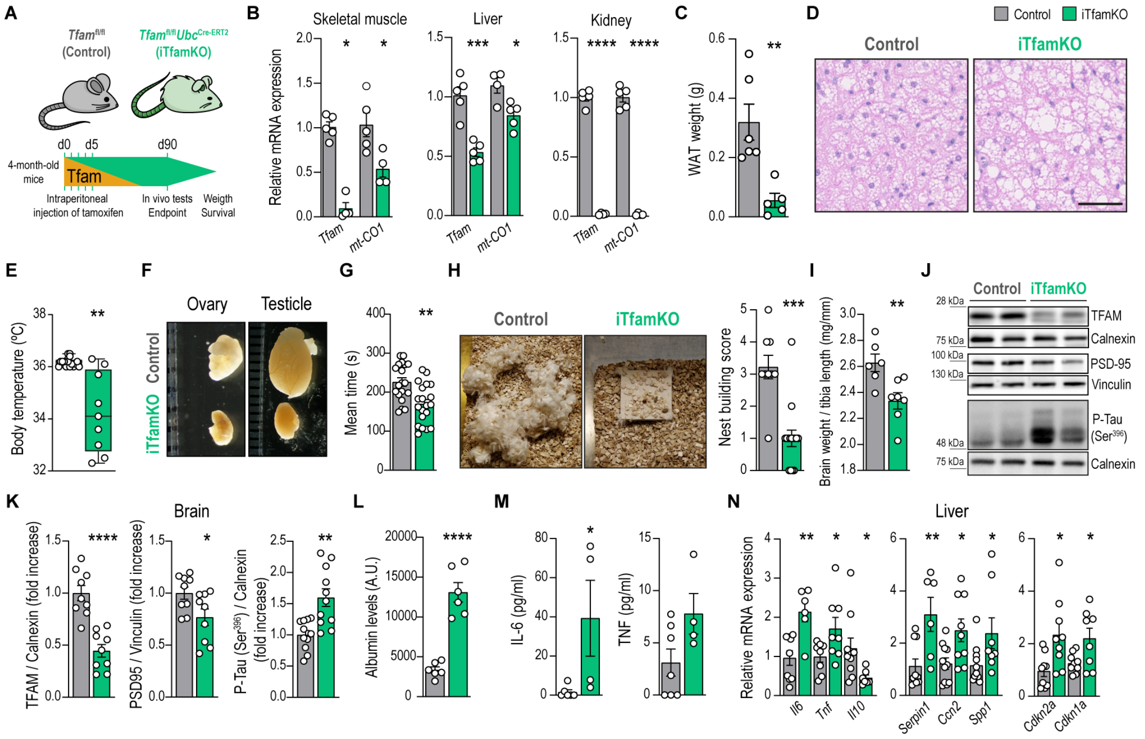

Figure S1. Inducible deletion of *Tfam* in adult mice precipitates multimorbidity. (**A**) Experimental design of tamoxifen inducible deletion of *Tfam* in adult mice. (**B**) Relative mRNA levels of *Tfam* in the skeletal muscle, liver, and kidney of control and iTfamKO mice (n = 4 to 5). (**C**) Quantification of gonadal white adipose tissue (WAT) weight (n = 5 to 6). (**D**) Representative hematoxylin and eosin–stained sections of brown adipose tissue. Scale bar: 50 µm. (**E**) Quantification of mouse body temperature (n = 9 to 16). (**F**) Representative picture of mouse gonads. (**G**) Rotarod test performance expressed as the mean time spent on the rotating rod in all trials combined (n = 19 to 20). (**H**) Representative picture of a mouse nest and its respective score (n = 3 to 5). (**I**) Quantification of brain weight normalized to tibia length (n = 6 to 7). (**J**) Representative immunoblot of TFAM, PSD-95, and phospho-Tau (Ser396) proteins in brain samples. (**K**) Densitometry analysis of brain immunoblots (n = 8 to 12). (**L**) Quantification of albumin levels in urine (n = 6). (**M**) Concentration of IL-6 and TNF in the serum (n = 4 to 7). (**N**) Relative mRNA levels of genes encoding inflammatory mediators (*Il6*, *Tnf*, *Il10*), pro-fibrotic factors (*Serpin1*, *Ccn2*, *Spp1*), and senescence-associated markers (*Cdkn2a*, *Cdkn1a*) in the liver (n = 6 to 11). All experiments were performed 90-days post-tamoxifen injection. Data are shown as means ± SEM, where each dot is a biological sample. P values were determined by (B, C, G, I, and K to N) unpaired two-tailed Student's t test, (E) Wilcoxon text or (H) two-tailed Mann-Whitney U test. *$P \leq 0.05$; **$P \leq 0.01$; ***$P \leq 0.001$; and ****$P \leq 0.0001$. Source data and exact P values are provided as a Source Data file.

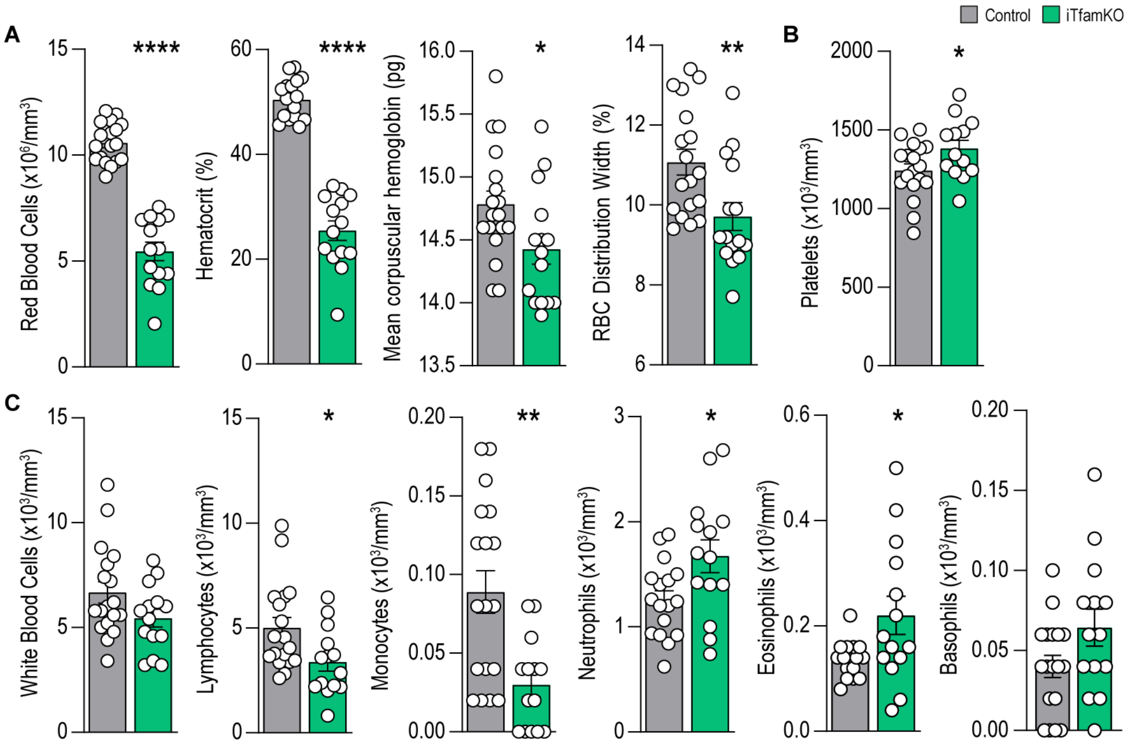

Figure S2. iTfamKO mice exhibit severe alterations in the hematopoietic cell compartment. (**A**) Quantification of hematologic parameters related to red blood cell (RBC) biology in control and iTfamKO mice (*n* = 14 to 18). (**B**) Quantification of blood platelets (*n* = 13 to 18). (**C**) Quantification of hematologic parameters related to white blood cell biology (*n* = 14 to 18). All experiments were performed 90-days post-tamoxifen injection. Data are shown as means ± SEM, where each dot is a biological sample. *P* values were determined by (A to C) unpaired two-tailed Student's *t* test. **P* ≤ 0.05; ***P* ≤ 0.01; and *****P* ≤ 0.0001. Source data and exact *P* values are provided as a Source Data file.

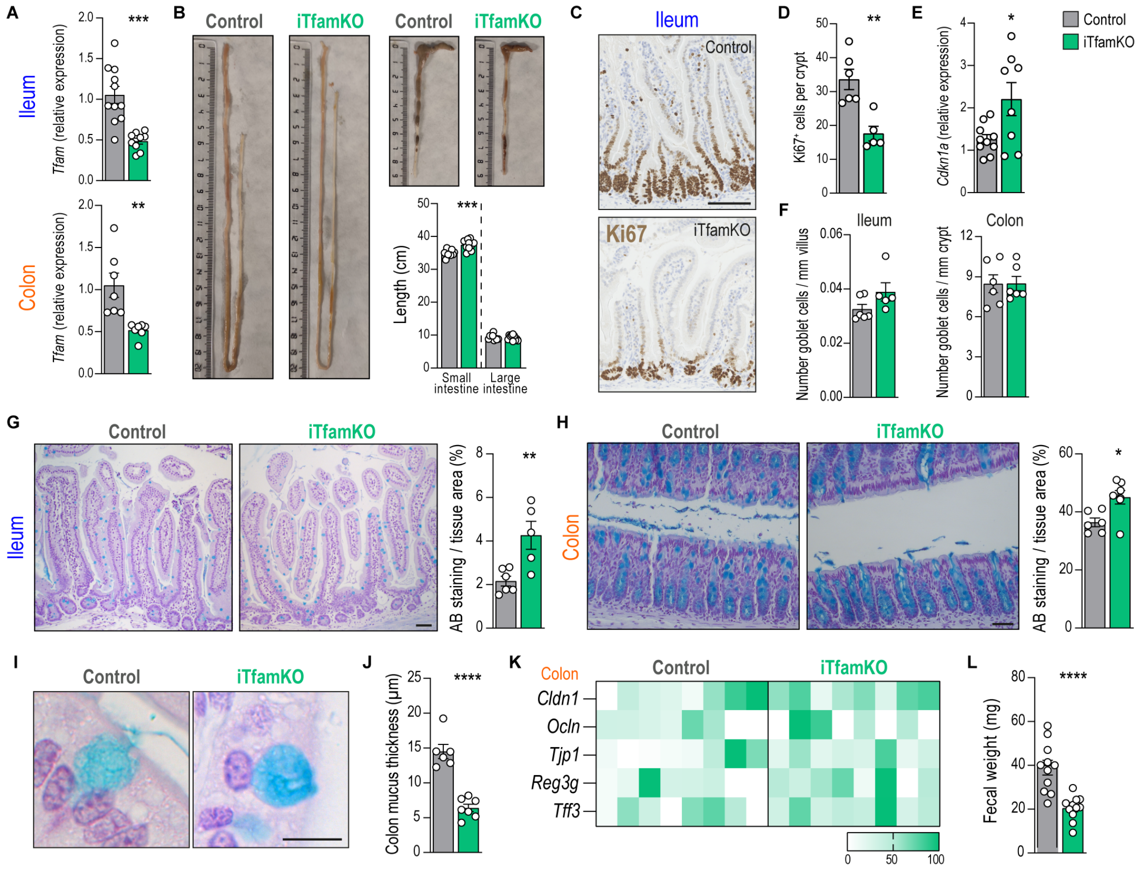

Figure S3. Analysis of intestinal barrier integrity in iTfamKO mice. (**A**) Relative expression levels of *Tfam* in the ileum and the colon of control and iTfamKO mice (n = 7 to 11). (**B**) Representative picture of the small and large intestines, and its respective length quantification (n = 9 to 10). (**C**) Representative immunohistochemistry staining of the cell proliferation marker Ki67 in the ileum. Scale bar: 125 μm. (**D**) Quantification of Ki67-positive cells per crypt in the ileum (n = 5 to 6). (**E**) Relative mRNA levels of the senescence-associated gene *Cdkn1a* in the ileum (n = 9 to 10). (**F**) Quantification of goblet cell number in the ileum and the colon (n = 5 to 6). (**G**) Representative Alcian blue–stained sections and quantification of stained area in the ileum (n = 5 to 6). Scale bar: 50 μm. (**H**) Representative Alcian blue–stained sections and quantification of stained area in the colon (n = 6 to 7). Scale bar: 50 μm. (**I**) Representative image of Alcian blue-positive goblet cells in the ileum. Scale bar: 10 μm. (**J**) Quantification of mucus layer thickness by Alcian blue staining in the colon (n = 6 to 7). (**K**) Heatmap depicting normalized values of expression levels of genes associated with tight junctions (*Cldn1*, *Ocln*, *Tjp1*), antimicrobial peptides (*Reg3g*), and barrier function (*Tff3*) in the colon assessed by qPCR (n = 7 to 9). (**L**) Quantification of feces weight (n = 11). All experiments were performed 90-days post-tamoxifen injection. Data are shown as means ± SEM, where each dot is a biological sample. P values were determined by (A, B, D to H, and J to L) unpaired Student's t test. *$P \leq 0.05$; **$P \leq 0.01$; ***$P \leq 0.001$; and ****$P \leq 0.0001$. Source data and exact P values are provided as a Source Data file.

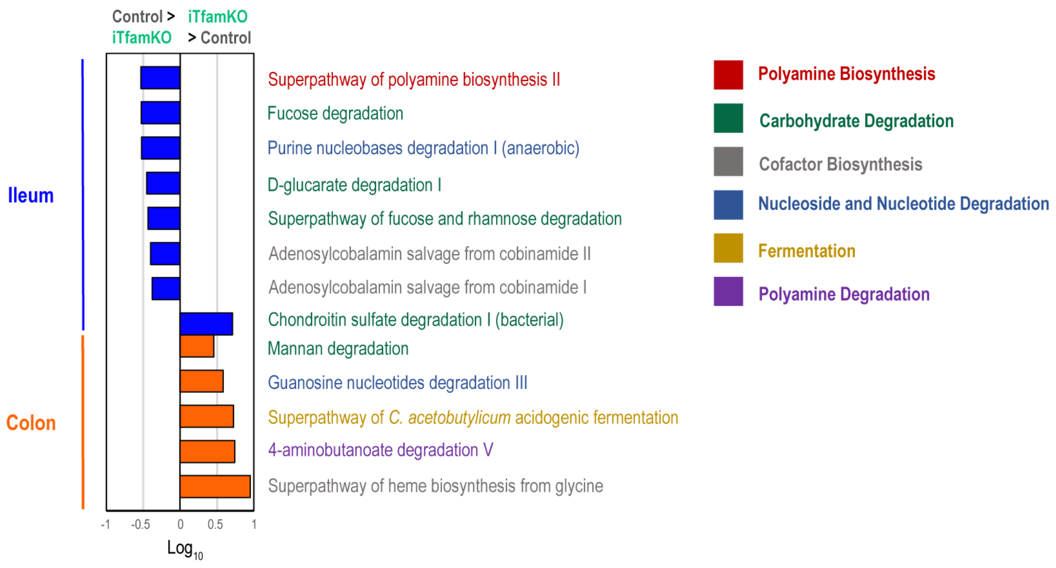

Figure S4. Predictive metabolic profiling of the intestinal microbiota in iTfamKO mice. PICRUSt2 prediction of metabolic pathways in ileal and colonic metagenomic data of control and iTfamKO mice (FDR, $Q < 0.05$; |SNR| > 0.5; |LDA| > -1; |fold change| > 2; maximal index > 1000). Source data are provided as a Source Data file.

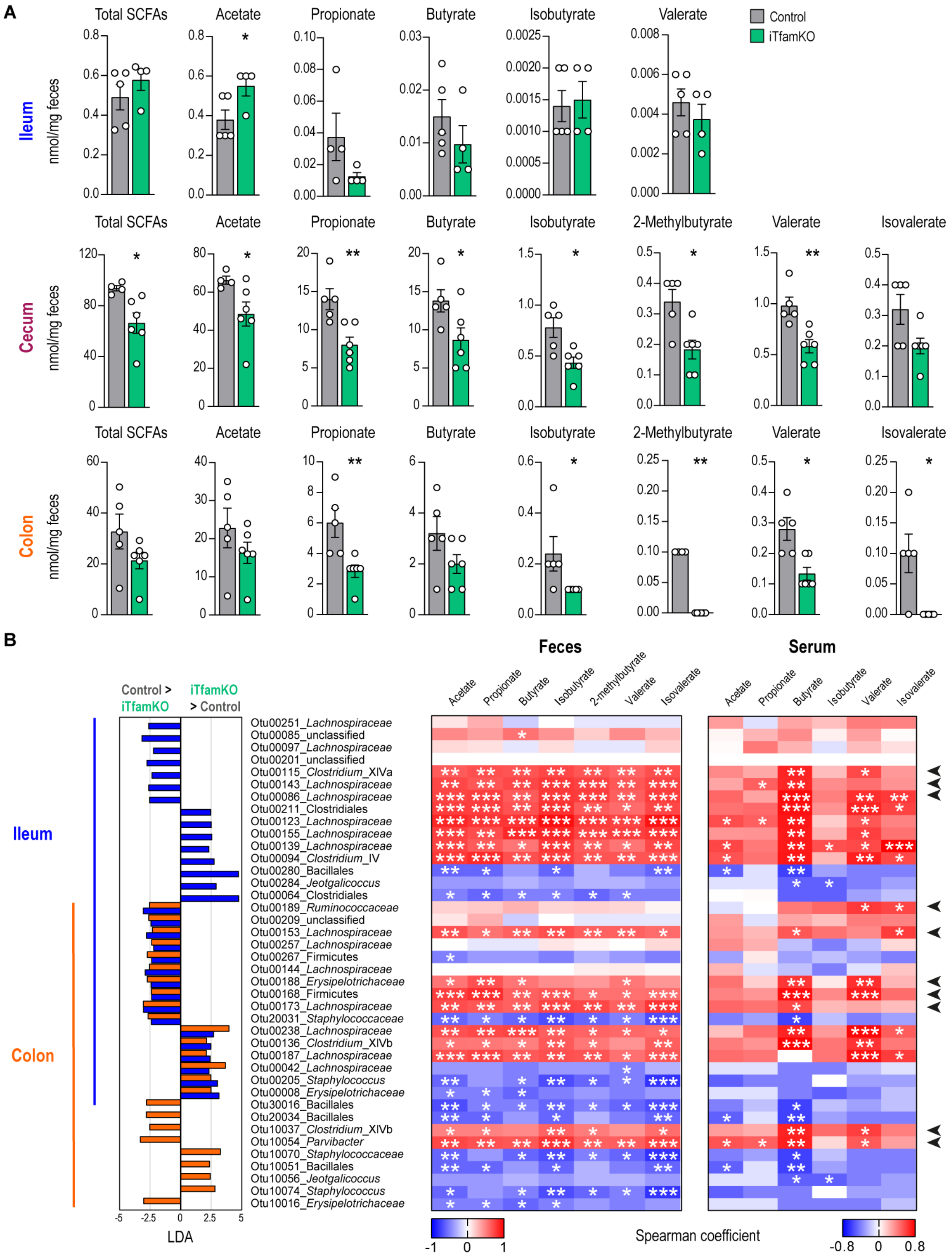

Figure S5. Evaluation of short-chain fatty acids (SCFAs) in the intestine of iTfamKO mice. (**A**) Quantification of SCFAs in the contents of the ileum, the cecum, and the colon of control and iTfamKO mice (*n* = 4 to 6). (**B**) Left: differentially abundant operational taxonomic units (OTUs) depicted with lineal discriminant analysis (LDA) values of linear discriminant effect size (LEfSe, $P < 0.05$; FDR, $Q < 0.05$; $|SNR| > 0.5$; $|LDA| > 2$; |fold change| > 10; maximal abundance > 0.001) comparing the ileum and colonic microbiota in control versus iTfamKO mice. Right: heatmap depicting Spearman's rank correlation coefficients between differentially abundant OTUs in ileal and colon-resident microbiota,

and the concentration of SCFAs in the feces and the serum of mice. All experiments were performed 90-days post-tamoxifen injection. Data are shown as means ± SEM, where each dot is a biological sample. *P* values were determined by (A) unpaired two-tailed Student's *t* test or two-tailed Mann-Whitney *U* test, or (B) PICRUSt2 software. $^{*}P \leq 0.05$; $^{**}P \leq 0.01$; and $^{***}P \leq 0.001$. Source data are provided as a Source Data file.

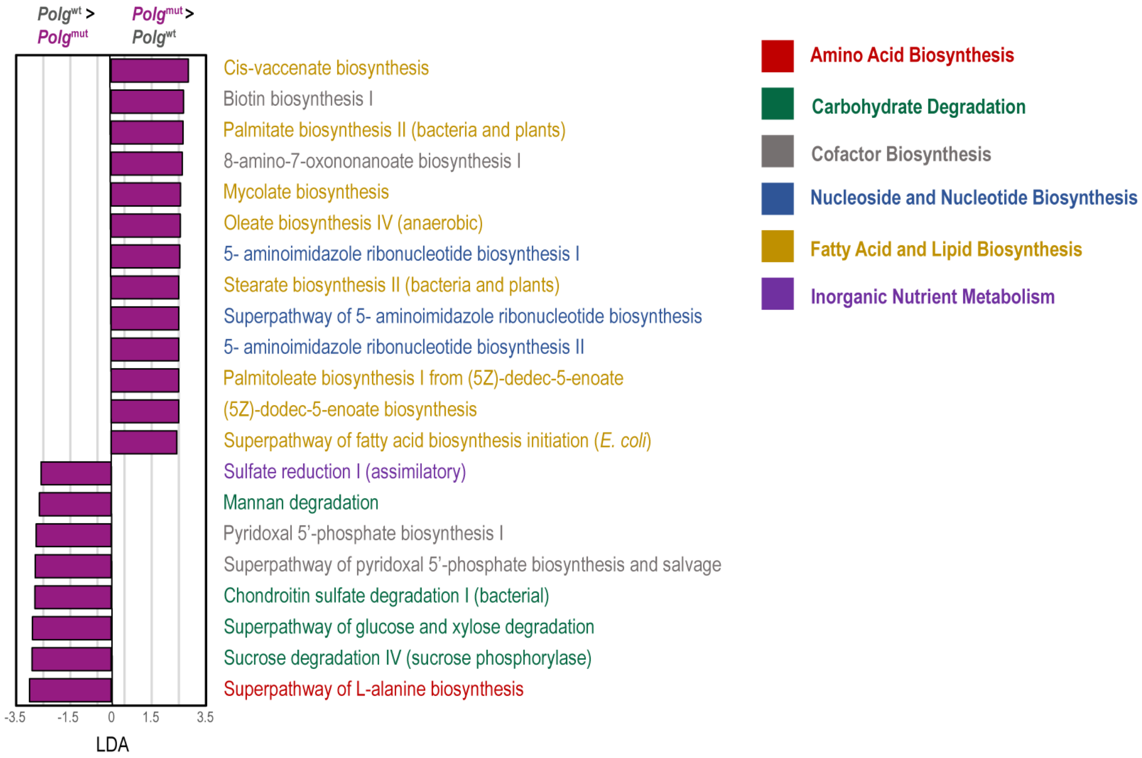

Figure S6. Predictive metabolic profiling of fecal microbiota in mtDNA-mutator mice. PICRUSt2 prediction of metabolic pathways in fecal metagenomic data of control and *Polg*^{mut} mice (FDR, $q < 0.05$; $|SNR| > 1$; $|LDA| > 2$). Source data are provided as a Source Data file.

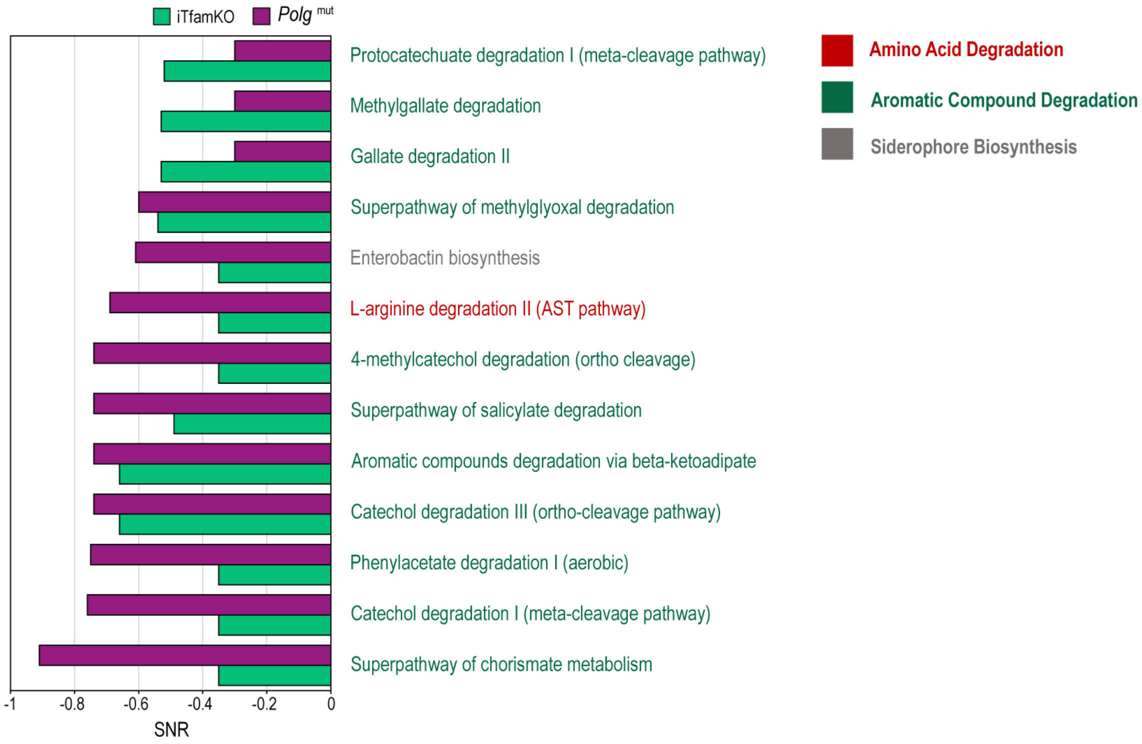

Figure S7. Predictive metabolic profile of intestinal microbiota shared by iTfamKO and mtDNA-mutator mice. PICRUSt2 prediction of shared metabolic pathways in metagenomic data of iTfamKO and *Polg*^{mut} mice (FDR, $q < 0.05$; $|SNR| > 0.2$; $|LDA| > -1$; $|fold\ change| > 1$; maximal abundance > 0). Source data are provided as a Source Data file.

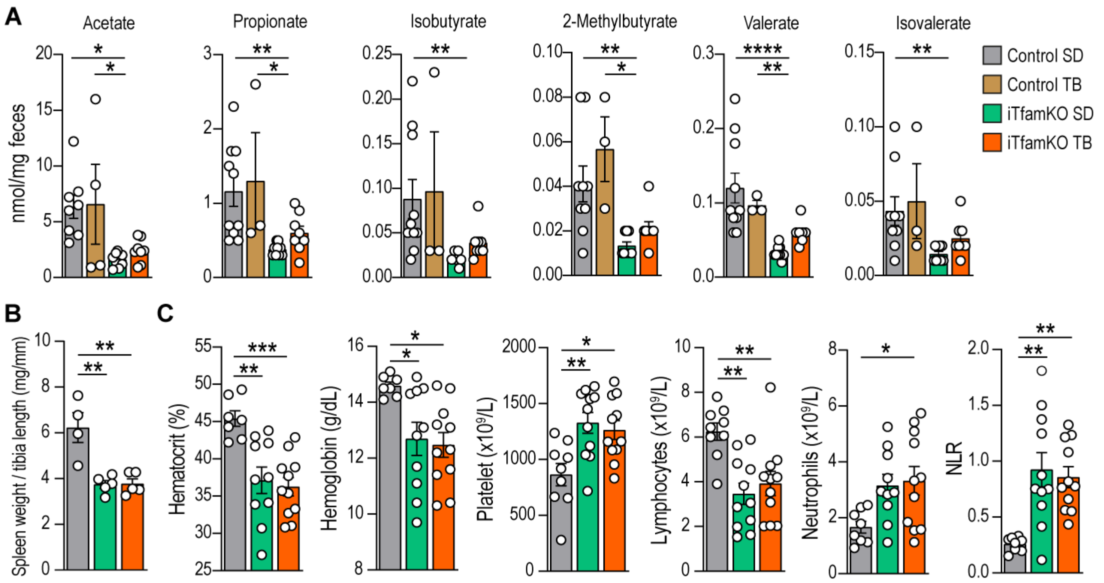

Figure S8. Analysis of short-chain fatty acids (SCFAs) and hematological parameters in iTfamKO mice after tributyrin administration. (**A**) Quantification of SCFAs in the feces of control and iTfamKO mice fed either a standard diet (SD) or a 10% tributyrin-supplemented diet (TB) (*n* = 3 to 10). (**B**) Quantification of spleen weight normalized to tibia length (*n* = 4 to 5). (**C**) Quantification of hematological parameters related to red blood cells, platelets, and white blood cells (*n* = 7 to 11). Data are shown as means ± SEM, where each dot is a biological sample. *P* values were determined by (A to C) one-way analysis of variance (ANOVA) with Tukey's multiple comparisons test. **P* ≤ 0.05; ***P* ≤ 0.01; ****P* ≤ 0.001; and *****P* ≤ 0.0001. Source data and exact *P* values are provided as a Source Data file.

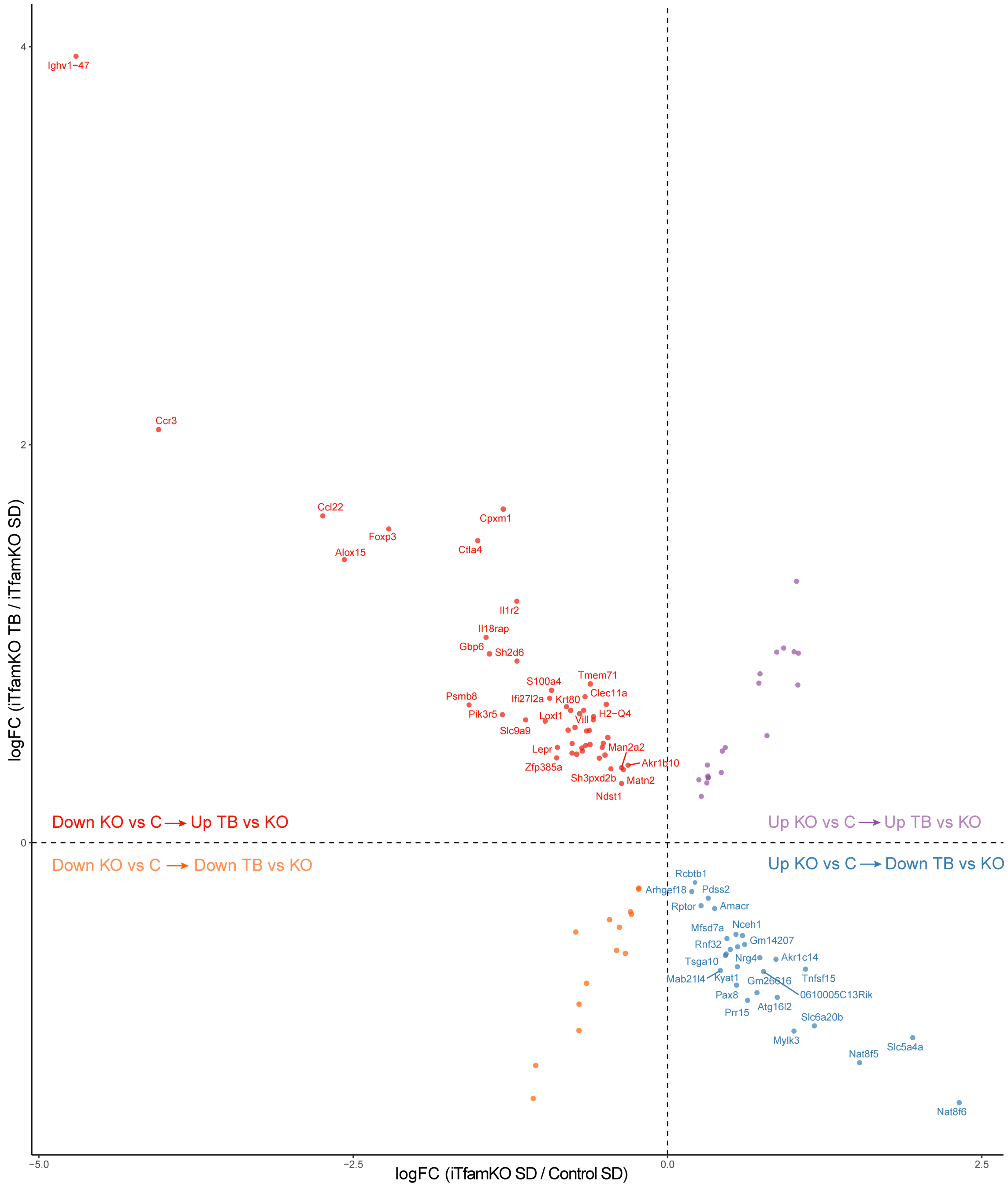

Figure S9. Differentially expressed genes in the ileum of iTfamKO mice after tributyrin administration. Four-quadrant scatter plot depicting differentially expressed genes in the ileum RNA-sequencing analysis of iTfamKO mice fed either a standard diet (SD) or a 10% tributyrin-supplemented diet (TB).

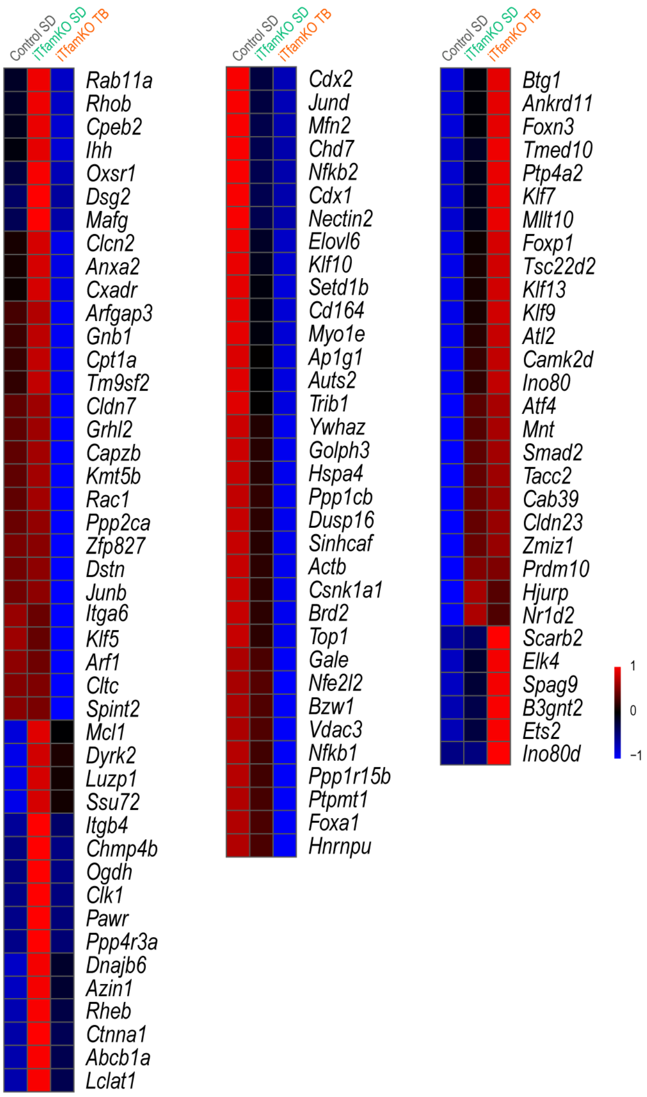

Figure S10. Expression of H3K27bu-dependet genes in the RNA-sequencing analysis of the ileum of iTfamKO mice after tributyrin administration. Heatmap depicting expression of H3K27bu-dependent genes in the ileum RNA-sequencing analysis of iTfamKO mice fed either a standard diet (SD) or a 10% tributyrin-supplemented diet (TB).

Table S1. Primers for qPCR analysis of mouse intestines.

Gene	Oligonucleotide (FW)	Oligonucleotide (RV)
Tfam	CAGGAGGCAAAGGATGATTC	CCAAGACTTCATTTCATTGTCG
Mt-CO1	CTCGCCTAATTTATTCCACTTCA	GGGGCTAGGGGTAGGGTTAT
Gdf15	CAACCAGAGCCGAGAGGAC	TGCACGCGGTAGGCTTC
Mthfd2	AGGTCCCAAGCCTTTGAGTT	GTAAGGGAGTGCCGTTGAAA
Cdkn1a	CTGACAGATTTCTATCACTCC	TTAAGACACACAGAGTGAGG
Cdkn2a	CCCGCCTTTTTCTTCTTAG	TTTCTCATGCCATTCCTTTC
Tnf	CTATGTCTCAGCCTCTTCTC	CATTTGGGAACTTCTCATCC
Il6	GTCTATACCACTTCACAAGTC	TGCATCATCGTTGTTCATAC
Il10	TGCCTTCAGCCAGGTGAAGACTTTC	CTTGATTTCTGGGCCATGCTTCTCTG
Serpine1	CCAACATCTTGGATGCTGAA	GCCAGGGTTGCACTAAACAT
Ccn2	AACTTCATGGATGGCCTCAAA	ACCCGGCTGCAGCTTGT
Spp1	ATGAGATTGGCAGTGATTTG	CATCCTTTTCTTCAGAGGAC
Cldn1	TCTACGAGGGACTGTGGATG	TCAGATTCAGCAAGGAGTCG
Ocln	GCTGTGATGTGTGTGAGCTG	GACGGTCTACCTGGAGGAAC
Tpj1	AGGACACCAAAGCATGTGAG	GGCATTCCTGCTGGTTACA
Defa5	CTCCTCTCTGCCCTTGTCCT	GATTTCTGCAGGTCCCAAAA
Defensin	GGTGATCATCAGACCCCAGCATCAGT	AAGAGACTAAAACTGAGGAGCAGC
Crypt1	TCAAGGGCTGCAAAGGAAGAGAAC	TGGTCTCCATGTTCAGCGACAGC
Lyz1	GAGACCGAAGCACCGACTATG	CGGTTTTGACATTGTGTTCGC
Ang4	GGTTGTGATTCCTCCAACTCTG	CTGAAGTTTTCTCCATAAGGGCT
Reg3g	ATGCTTCCCCGTATAACCATCA	GGCCATATCTGCATCATACCAG
Tff3	TTGCTGGGTCCTCTGGGATAG	TACACTGCTCCGATGTGACAG
Hprt	TCCTCCTCAGACCGCTTTT	CCTGGTTCATCATCGCTAATC
Pp1a	ACGCCACTGTCGCTTTTC	GCAAACAGCTCGAAGGAGAC
B2m	TACATACGCCTGCAGAGTTAAGCA	TGATCACATGTCTCGATCCCAG

Table S2. Optimization of multiple reaction monitor (MRM) condition acquisition modes by direct infusion of 3-nitrophenylhydrazine (3NPH) derivatives.

**3NPH-SCFA derivatives**	**Precursor ion (m/z)**	**Product ion (m/z)**	**Fragmentor voltage (V)**	**Collision energy (eV)**
AA (C2)	194.1	**137**<br>46	92	**16**<br>44
PA (C3)	208.1	**137**<br>46	92	**20**<br>48
i-BA/BA (C4)	222.1	**137**<br>107	92	**20**<br>28
2-Me-BA/i-VA/VA (C5)	236.1	**137**<br>107	102	**20**<br>32
2-Me-VA (C6)	250.1	**137**<br>107	102	**20**<br>31

Uncropped scans of blots shown in Supplementary Figures.

Relative to Fig. S1

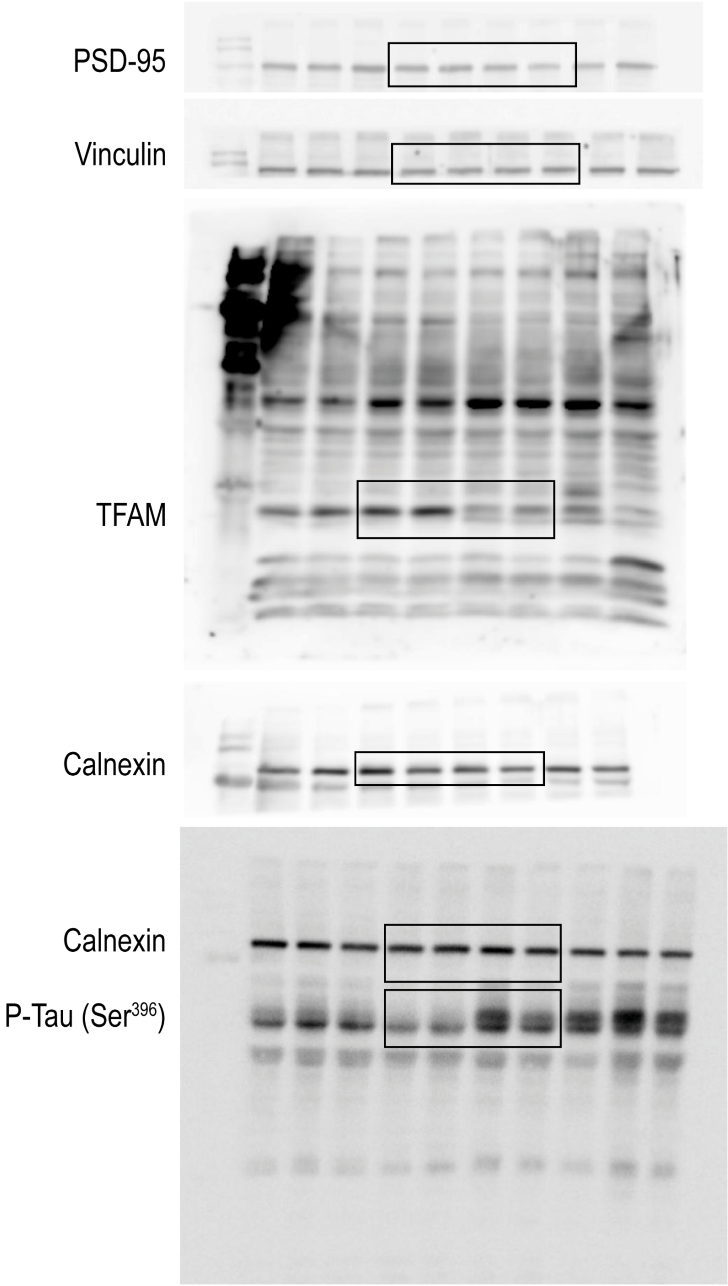

Supplement: Supplementary file 1 — Supplementary Information file [file 41467_2026_70547_MOESM1_ESM.pdf]
