# Supplementary material for: Butyrate extends health and lifespan in mice with mitochondrial deficiency
Source: Nat Commun. 2026 Mar 13;17:3909. doi: 10.1038/s41467-026-70547-4 (PMC13129094; doi:10.1038/s41467-026-70547-4)
Supplement: Supplementary file 2 — Description of Additional Supplementary Files [file 41467_2026_70547_MOESM2_ESM.pdf]

Description of Additional Supplementary Files

Supplementary Data: uncropped scans of gels and blots shown in Figs. 1P, 6H, 7A, and 7C.
