# Supplementary material for: Butyrate extends health and lifespan in mice with mitochondrial deficiency
Source: Nat Commun. 2026 Mar 13;17:3909. doi: 10.1038/s41467-026-70547-4 (PMC13129094; doi:10.1038/s41467-026-70547-4)

Uncropped scans of gels and blots shown in Figures.

Relative to Fig. 1P

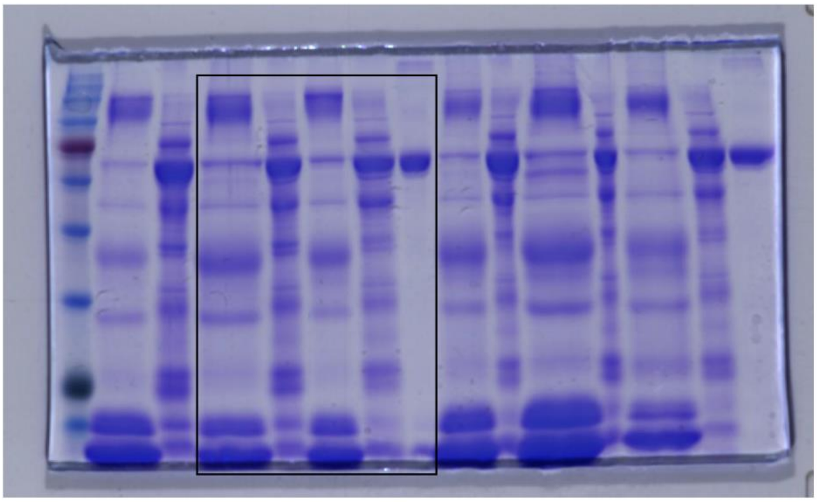

Relative to Fig. 6H

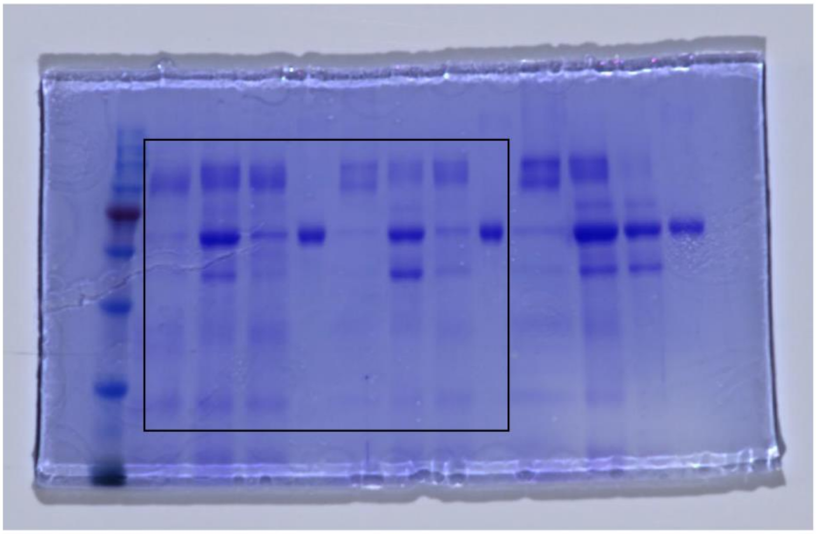

Relative to Fig.7A

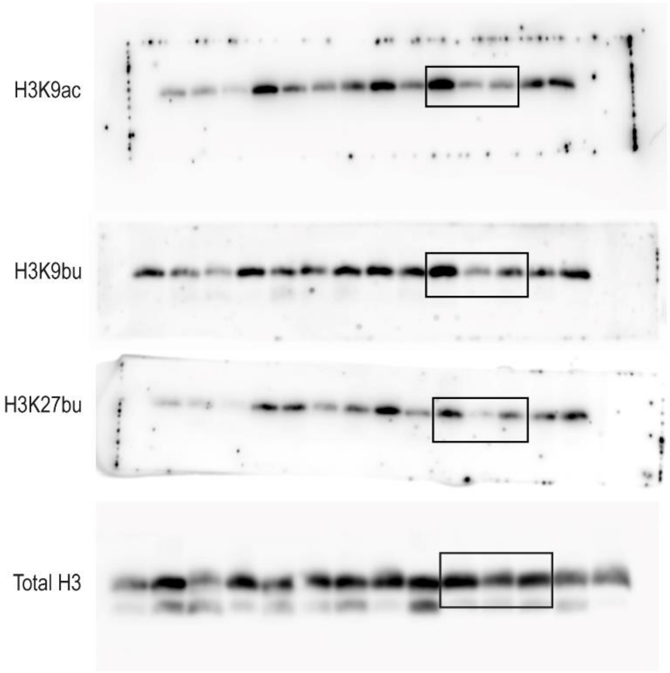

Relative to Fig.7C

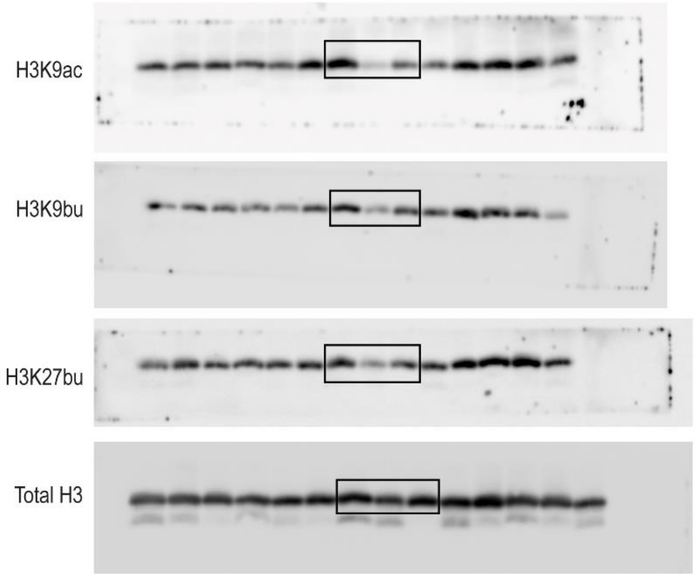

Supplement: Supplementary file 3 — Supplementary Data [file 41467_2026_70547_MOESM3_ESM.pdf]
