# Supplementary material for: Butyrate extends health and lifespan in mice with mitochondrial deficiency
Source: Nat Commun. 2026 Mar 13;17:3909. doi: 10.1038/s41467-026-70547-4 (PMC13129094; doi:10.1038/s41467-026-70547-4)
Supplement: Supplementary file 4 — Reporting Summary [file 41467_2026_70547_MOESM4_ESM.pdf]

nature portfolio

Corresponding author(s): María Mittelbrunn

Last updated by author(s): 20/02/2026

Reporting Summary

Nature Portfolio wishes to improve the reproducibility of the work that we publish. This form provides structure for consistency and transparency in reporting. For further information on Nature Portfolio policies, see our Editorial Policies and the Editorial Policy Checklist.

Statistics

For all statistical analyses, confirm that the following items are present in the figure legend, table legend, main text, or Methods section.

n/a	Confirmed	
☐	☒	The exact sample size (*n*) for each experimental group/condition, given as a discrete number and unit of measurement
☐	☒	A statement on whether measurements were taken from distinct samples or whether the same sample was measured repeatedly
☐	☒	The statistical test(s) used AND whether they are one- or two-sided<br>*Only common tests should be described solely by name; describe more complex techniques in the Methods section.*
☒	☐	A description of all covariates tested
☐	☒	A description of any assumptions or corrections, such as tests of normality and adjustment for multiple comparisons
☐	☒	A full description of the statistical parameters including central tendency (e.g. means) or other basic estimates (e.g. regression coefficient) AND variation (e.g. standard deviation) or associated estimates of uncertainty (e.g. confidence intervals)
☐	☒	For null hypothesis testing, the test statistic (e.g. *F*, *t*, *r*) with confidence intervals, effect sizes, degrees of freedom and *P* value noted<br>*Give P values as exact values whenever suitable.*
☒	☐	For Bayesian analysis, information on the choice of priors and Markov chain Monte Carlo settings
☒	☐	For hierarchical and complex designs, identification of the appropriate level for tests and full reporting of outcomes
☒	☐	Estimates of effect sizes (e.g. Cohen's *d*, Pearson's *r*), indicating how they were calculated

Our web collection on statistics for biologists contains articles on many of the points above.

Software and code

Policy information about availability of computer code

Data collection	NA
Data analysis	Statistical analyses were performed using GraphPad Prism 9 or Past 3.22 software. 16s RNA libraries were then sequenced using an Illumina MiSeq instrument, and sequences were curated and analyzed using the mothur (v.1.40.5) software package.<br>cDNA libraries were prepared from total RNA and then, validated and quantified by an Agilent 4200 TapeStation in Haplox. After passing library inspection, stranded mRNA was sequenced either on the Illumina Novaseq Xplus platform, and FastQ files were generated containing nucleotide data and quality scores for each position. The quality of FastQ files was checked using FastQC (v0.11.9) RNA-sequencing reads were mapped to the Mus musculus reference genome GRCm39 using either Hisat2 (v2.2.1) or STAR (v2.5.2) software. Reads were then pre-processed with SAMtools (v1.13) to transform Sequence Alignment/Map files into Binary Alignment/Map files and sorted. The number of reads covered by each gene was calculated by HTSeq-Count (v1.99.2).<br>Downstream data analysis was performed with R (v4.3.2). Differential gene expression (DEG) analysis was performed using DESeq2 (v1.44). Genes with P value < 0.05 and |log2-fold change| > 0.6 were determined to show statistically significant differences in group comparison. Over-representation analysis (ORA) and gene set enrichment analysis (GSEA) were performed using clusterProfiler (v4.8.3) package in GO, KEGG, WikiPathways, Reactome and the Hallmarks of the Molecular signatures databases. PCA plots, chord diagrams and heatmaps were visualized by using ggplot2 (v3.4.4), circlize (v0.4.15) and pheatmap (v1.0.12), respectively.<br>Images were analysed with ImageJ software (v1.53n).

For manuscripts utilizing custom algorithms or software that are central to the research but not yet described in published literature, software must be made available to editors and reviewers. We strongly encourage code deposition in a community repository (e.g. GitHub). See the Nature Portfolio guidelines for submitting code & software for further information.

Data

Policy information about availability of data

All manuscripts must include a data availability statement. This statement should provide the following information, where applicable:

- Accession codes, unique identifiers, or web links for publicly available datasets
- A description of any restrictions on data availability
- For clinical datasets or third party data, please ensure that the statement adheres to our policy

Microbiota 16S rRNA gene sequencing data are publicly available via NCBI with
BioProject number PRJNA1291203.

RNA-seq data on Ileum data are publicly available via NCBI with
BioProject number PRJNA1301342 (GSE304464).

Research involving human participants, their data, or biological material

Policy information about studies with human participants or human data. See also policy information about sex, gender (identity/presentation), and sexual orientation and race, ethnicity and racism.

Reporting on sex and gender	NA
Reporting on race, ethnicity, or other socially relevant groupings	NA
Population characteristics	NA
Recruitment	NA
Ethics oversight	NA

Note that full information on the approval of the study protocol must also be provided in the manuscript.

Field-specific reporting

Please select the one below that is the best fit for your research. If you are not sure, read the appropriate sections before making your selection.

☒ Life sciences ☐ Behavioural & social sciences ☐ Ecological, evolutionary & environmental sciences

For a reference copy of the document with all sections, see nature.com/documents/nr-reporting-summary-flat.pdf

Life sciences study design

All studies must disclose on these points even when the disclosure is negative.

Sample size	No statistical method was used to predetermine sample size, but a minimum of four samples were used per experimental group and condition. Four samples were considered as the minimun to reduce the number of animals and have enough information for statistical tests. In addition, some experiments were performed with more mice because they were initially caged together.
Data exclusions	Outliers were identified by the ROUT method (5%) and removed.
Replication	Representative plots and graphs summarize results of at least two independent experiments
Randomization	Mice were grouped by their date of birth for this study.
Blinding	Most of the investigators were blided in the experiments performed. However, in some experiments the appearence of the animal is a strong indicative of their group.

Reporting for specific materials, systems and methods

We require information from authors about some types of materials, experimental systems and methods used in many studies. Here, indicate whether each material, system or method listed is relevant to your study. If you are not sure if a list item applies to your research, read the appropriate section before selecting a response.

Materials & experimental systems

n/a	Involved in the study
☐	☒ Antibodies
☒	☐ Eukaryotic cell lines
☒	☐ Palaeontology and archaeology
☐	☒ Animals and other organisms
☒	☐ Clinical data
☒	☐ Dual use research of concern
☒	☐ Plants

Methods

n/a	Involved in the study
☒	☐ ChIP-seq
☒	☐ Flow cytometry
☒	☐ MRI-based neuroimaging

Antibodies

Antibodies used

anti-NeuN (1:500 dilution; Millipore, MAB377), anti-Ki67
832 antibody (RD Systems, AF3667), anti-TFAM (1:1000 dilution; Proteintech, 22586),
852 rabbit anti-calnexin (1:10000 dilution; Abcam, ab22595), rabbit anti-tau-P Ser 396
853 (1:1000 dilution; Life Technologies, 44752G), mouse anti-PSD95 (1:1000 dilution; BD
854 Biosciences, 610495) or mouse anti-vinculin (1:10000 dilution; Abcam, ab129002) for
855 brain lysates, or rabbit anti-H3K9ac (1:5000 dilution; Abcam, ab4441), rabbit anti-
856 H3K9bu (1:500 dilution; PTMBio, PTM-305), rabbit anti-H3K27bu (1:1000 dilution;
857 Merck, ABE2854), or rabbit anti-H3 (1:1000 dilution; Abcam, ab1791)

Validation

All the antibodies used were purchased to suppliers. The antibodies were validated by the supplier and previously published papers

Animals and other research organisms

Policy information about studies involving animals; ARRIVE guidelines recommended for reporting animal research, and Sex and Gender in Research

Laboratory animals

Most of the experiments were performed with females due to the possibility to recage them but most findings have been replicated in males.

Wild animals

The study did not involve wild animals.

Reporting on sex

Findings apply to both sexes of mice.

Field-collected samples

The study did not involve field-collected samples.

Ethics oversight

All the procedures with animals were previously evaluated and approved (PROEX 287/16 and PROEX 52.1/23) by the Ethics commitee on animal experimentation of the CBMSO, the authorized commitee of the Spanish National Research Council or the Universidad Autónoma de Madrid and the regional government (Comunidad de Madrid)

Note that full information on the approval of the study protocol must also be provided in the manuscript.

Plants

Seed stocks

NA

Novel plant genotypes

NA

Authentication

NA
